# Identification of novel genetic variants associated with short stature in a Baka Pygmies population

**DOI:** 10.1007/s00439-020-02191-x

**Published:** 2020-06-24

**Authors:** Matteo Zoccolillo, Claudia Moia, Sergio Comincini, Davide Cittaro, Dejan Lazarevic, Karen A. Pisani, Jan M. Wit, Mauro Bozzola

**Affiliations:** 1 San Raffaele Telethon Institute for Gene Therapy (SR-Tiget), IRCCS San Raffaele Scientific Institute, Milan, Italy; 2 Department of Biology and Biotechnology “Lazzaro Spallanzani”, Università Degli Studi Di Pavia, Pavia, Italy; 3 Center for Omics Sciences, IRCCS San Raffaele Scientific Institute, Milan, Italy; 4 Pediatrics, Leiden University Medical Center, 2300 RC Leiden, Netherlands; 5 University of Pavia, and Onlus Il Bambino E Il Suo Pediatra, Via XX Settembre 28, Galliate, 28066 Novara, Italy

## Abstract

Human growth is a complex trait determined by genetic factors in combination with external stimuli, including environment, nutrition and hormonal status. In the past, several genome-wide association studies (GWAS) have collectively identified hundreds of genetic variants having a putative effect on determining adult height in different worldwide populations. Theoretically, a valuable approach to better understand the mechanisms of complex traits as adult height is to study a population exhibiting extreme stature phenotypes, such as African Baka Pygmies. After phenotypic characterization, we sequenced the whole exomes of a cohort of Baka Pygmies and their non-Pygmies Bantu neighbors to highlight genetic variants associated with the reduced stature. Whole exome data analysis revealed 29 single nucleotide polymorphisms (SNPs) significantly associated with the reduced height in the Baka group. Among these variants, we focused on SNP rs7629425, located in the 5′-UTR of the Hyaluronidase-2 (*HYAL2*) gene. The frequency of the alternative allele was significantly increased compared to African and non-African populations. In vitro luciferase assay showed significant differences in transcription modulation by rs7629425 C/T alleles. In conclusion, our results suggested that the *HYAL2* gene variants may play a role in the etiology of short stature in Baka Pygmies population.

**Electronic supplementary material:**

The online version of this article (10.1007/s00439-020-02191-x) contains supplementary material, which is available to authorized users.

## Introduction

Human growth and, in particular, adult height are undoubtedly multifactorial processes, involving genetic, hormonal, nutritional and other environmental factors (Waldman and Chia 2013). Regulation of human adult stature has been of particular interest to geneticists, evolutionary and cultural anthropologists, as well as to pediatricians focused on growth disorders (Lettre 2011). Adult height is a prime example of a highly polygenic complex trait with a relatively high hereditability (≥ 69%) (Pemberton et al. 2018; Sohail et al. 2019). Polygenic traits are known to evolve differently from monogenic ones, through slight but coordinated shifts in the frequencies of a large number of alleles, each with a small effect (Sohail et al. 2019). Because a major proportion of adult stature is dependent upon an intact growth hormone (GH) and insulin-like growth factor I (IGF-I) axes, much attention in previous studies has been devoted to abnormalities related to these growth factor patterns. GH, GH-binding protein (GHBP) and IGF-I are among the key molecules involved in human growth and their abnormal secretion is often found in human growth disorders (Wit et al. 2016; Andradeet al. 2017). However, because of the phenotypic complexity, variants in components of the GH/IGF-I axes can only explain a small part of the variability of normal human growth (Durand and Rappold 2013). Indeed, recent genome-wide association studies (GWAS) identified approximately 700 common variants with a putative effect on determining adult height (Wood et al. 2014; Andrade et al. 2017).

From a clinical point of view, the term idiopathic short stature (ISS) is adopted when no recognizable cause of growth impairment is found despite an adequate diagnostic workup (Cohen 2014). Essentially, ISS is characterized by a height more than two standard deviations (SD) below the mean for sex and age without other clinical features in a child born with a normal birth size, encompassing familial short stature and constitutional delay of growth (Wit et al. 2008). Patients with ISS show a normal GH secretion in response to provocative stimuli. However, mean serum levels of IGF-I and GHBP are below the population average, suggesting partial insensitivity to GH (Wit et al. 2008; Kang 2017). Remarkably, a similar insensitivity to IGF-1 was scored in African Pygmies populations (Bozzola et al. 2009). African Pygmies, together with some other populations spread around the world, now commonly known as pygmoid populations, are characterized by an adult short stature, with males exhibiting an average height of about 150 cm or less in conjunction with reasonably well-conserved body proportions (Migliano et al. 2007; Meazza et al. 2011; Verdu 2016).

African Pygmies live in equatorial rain forest and share an economy based on hunting and gathering, exhibiting characteristic culture and behavior features (Le Bouc 2017). Genetic studies indicate a quite clear distinction between Pygmies and non-Pygmies populations (Verdu et al. 2009). Pygmies populations are distributed across equatorial Africa in two main clusters: one in East Africa (e.g., Uganda) including the Batwa and Efe groups, and the other one in West Africa (e.g., Cameroon) including the Kola and the Baka populations. Substantial admixtures between Pygmies and non-Pygmies may have occurred for a long time (Patin et al. 2014). However, the degree of admixture varies in a same region, e.g., Kola Pygmies from Southwest Cameroon show a relatively higher level of admixture compared to Baka Pygmies from Southeast Cameroon (Verdu et al. 2009). Despite this fact, genetic studies indicate a quite clear distinction between Pygmies and non-Pygmies (Verdu et al. 2009; Patin et al. 2014).

Over the years, several evolutionary hypotheses have been proposed to explain the short stature of Pygmies (Perry and Dominy 2009). These included the adaptation to food scarcity, difficulties of thermoregulation in the dense tropical forest with warm and humid conditions and trade-off between growth cessation and age at first reproduction caused by a high mortality rate (Bailey 1991; Perry et al. 2014). However, in recent years, the limitations of physiological data as well as scarce demographic, epidemiological, and paleo-anthropological evidence have led to the use of genetics approaches (Becker et al. 2011), including Whole Exome Sequencing (WES), to identify the genetic determinants influencing Pygmies’ short stature. Using high-density single nucleotide polymorphism (SNP) chip data, several studies of population genetics studies have found candidate chromosomal regions associated with short stature, including genes encoding for factors involved in the IGF-I axis, the iodine-dependent thyroid hormone and the bone homeostasis/skeletal remodeling pathways (Jarvis et al. 2012; Hsieh et al. 2016; Mendizabal et al. 2012). Lachance and collaborators (2012) searched for signals of positive selection in five high-coverage Western African Pygmies genomes and suggested that short stature may be due to selection of genes involved in development of the anterior pituitary gland, as well as in the crosstalk between the adiponectin and insulin-signaling pathways.

In the present study, we describe a WES analysis based on phenotypically characterized Baka Pygmies and Bantu non-Pygmies subjects, with the aim to identify potential genetic variations associated with the Pygmies’ short stature. Based on these results, we suggest that a variant of the *HYAL2* gene may have a role in the determination of short height in the Baka Pygmies population.

## Materials and methods

### Sample collection and genotyping scheme

Blood samples were collected from 84 Pygmies (35 males and 49 females) and 20 Bantu (6 males and 14 females) subjects, in serum-separator tubes and in Tempus Blood RNA tubes (ThermoFisher Scientific, Waltham, MA, USA). All samples were maintained at refrigerated temperature for 3 days, then frozen for transport and stored at – 20 °C until DNA extraction. Subjects were orally informed and those who gave their consent underwent clinical evaluation and blood withdrawal for genetic studies. The criteria for the enrolment of the Bantu control population were matched for age and sex, sympatry and clinical exclusion of phenotypically apparent diseases.

The genotype scheme considered initially 84 blood samples from Pygmies and 20 from Bantu individuals. Auxological data (weight, height, BMI) were available for 27 Pygmies and 20 Bantu, respectively. WES was then performed on a representative selection of eight Pygmies and five Bantu, at the lower or upper limits of the height distributions of these populations, respectively. The most significant genetic candidates WES results were then finally validated on a cohort of 76 Pygmies and 15 Bantu individuals.

### WES and mutation analysis

DNA were extracted from blood samples using QIAamp DNA Blood Mini Kit (Qiagen, Hilden, Germany) protocol, followed by qualitative and quantitative analyses by Quibit fluorimeter (ThermoFisher Scientific). Exome enrichment library preparation was performed using TruSeq DNA Exome (Illumina, San Diego, CA, USA). Sequencing was done using HiSeq2000 (Illumina), based on SBS chemistry. We generate PE (pair end) reads, 100 nucleotides long, to obtain in average a 20–30 × coverage. Sequence reads were mapped to the human reference genome GRCh37-9/hg19 using the Burrows-Wheeler Aligner v.1 (Li and Durbin 2010). Variant calling was performed using Free Bayes pipeline (Garrison and Marth 2012) and haplotype-based variant detector from short-read sequencing, with the following settings: minimum mapping quality = 30, minimum base quality = 20, minimum supporting allele = 0, genotype variant threshold = 0: QUAL ≥ 30; total read depth at the locus ≥ 5. Each variant was annotated using databases dbSNP-138, dbNSFP2.4, hg19.refGene, OMIM, HAPMAP, and 1000 Genome. Functional annotation was performed by SnpEff algorithm (https://snpeff.sourceforge.net).

The pairwise genetic difference was estimated for all populations by calculating Wright’s F statistics (F_st_) (Wright 1951). Data sets used for F_st_ estimation were derived from the 1000 Genome database with the following adjustments: African without Americans of African Ancestry and European without Finnish in Finland, East Asian without Vietnam, and Northern and Western Ancestry.

Differences in genotype frequencies associated with different phenotypes were tested for each autosomal biallelic variant selected by F_st_, with sparse Partial Least Squares regression (sPLS) (Chun and Keleş 2010). For sPLS, the number of components included in the model was set to two. The number of variables kept for the first component, which determines the strength of the variant selection, was set to 400.

### Sequence validation

The Fluidigm 48 × 48 Access Array IFC system (Fluidigm, San Francisco, CA, USA) was used to validate 46 variants identified by WES and associated with short stature in Baka Pygmies. In detail, specific primer pairs (sequences available upon request) were designed to amplify flanking regions of each variant. Then universal primers sequences (5′-ACACTGACGACATGGTTCTACA and 5′-TACGGTAGCAGAGACTTGGTCT) were ligated at the 5′ termini to all PCR products. The amplification PCRs were performed on 76 Pygmy and 15 Bantu subjects using 50 ng of genomic DNA, 1 × FastStart High-Fidelity Reaction Buffer with MgCl_2_, 5% DMSO (v/v), dNTPs (200 µM each), universal primers (1 µM each), 1 × Access Array loading reagent and FastStart High-Fidelity Enzyme Blend (Roche, Basel, Switzerland). Subsequently, thermal cycling on a Fluidigm FC1 Cycler was performed according to the manufactures’ condition. Sanger sequencing analyses for rs7629425 variant were performed on 10 ng of genomic DNA using the following primers: (5′-AAAGGCATTCAGGTCCAGTG; 5′-AATAAGCAGGTGTTTGGGGA).

### Generation of pCMV-Luc, pCMV-HYAL2-WT, and pCMV-HYAL2-MUT plasmids

pCMV-Luc plasmid was generated from firefly pGL3-derived luciferase gene *Hind*III and *BamH*I cloning into pcDNA 3.1 vector (Invitrogen, Carlsbad, CA, USA). Starting from pCMV-Luc, two DNA fragments of 50 nucleotides of 5′-UTR of *HYAL2* gene containing either rs7629425 reference (C) or alternative alleles (T) were amplified using a three-round PCR and then cloned into pCMV-Luc, adjacent to the firefly luciferase gene, thus originating plasmids pCMV-HYAL2-WT and pCMV-HYAL2-MUT, respectively (Suppl. Figure 1). In the first PCR, the firefly luciferase gene, including the restriction site for *BamH*I, was amplified from pCMV-Luc together with a small sequence containing the last 20 nucleotides of the 5′-UTR of *HYAL2* variants using these primer pairs (i.e., 1 for reference or two for alternative allele). The second PCR using primers four and three amplified the first PCR amplicons adding the full portion of interest of the 5-’UTR as well as the *Hind*III restriction site; the last PCR using primers four and five added random nucleotides upstream of *Hind*III and *BamH*I to improve next restriction digestion’s efficiency. All primers sequences are reported in Supplementary Table 3. PCRs were performed using the following thermal profile: 98 °C for 3 min; 98 °C for 20 s, 55 °C for 40 s, 72 °C for 60 s (five cycles); 98 °C for 20 s, 65 °C for 40 s, 72 °C for 60 s (20 cycles); final extension at 72 °C for 10 min.

Following purification with Agencourt AMPure XP beads (Beckman Coulter, Brea, CA, USA), analysis with either Agilent DNA High-Sensitivity Kit for BioAnalyzer (Agilent, Santa Clara, CA, USA) or 0.8% agarose gel electrophoresis and validation with Sanger sequencing on both DNA strands, PCR products and pCMV-Luc vector were digested with *Hind*III and *BamH*I enzymes (New England Biolabs, Ipswich, MA, USA). PCR fragments were gel-purified and cloned into digested pCMV-Luc vector thus giving pCMV-HYAL2-WT (containing rs7629425 C allele) or pCMV-HYAL2-MUT (containing T allele) plasmids. Following heat-shock transformation in *E.coli* One-Shot Top10 (Invitrogen) competent cells, colony PCR was performed to select positive clones, then plasmids were extracted using Promega Wizard Plus SV Minipreps DNA Purification System (Promega, Madison, WI, USA). Recombinant clones, containing pCMV-HYAL2-WT and pCMV-HYAL2-MUT plasmids, were verified by Sanger sequencing using primers Luc1 and Luc3 (Eurofins GATC Biotech, Germany).

### Transfection of pCMV-Luc, pCMV-HYAL2-WT and pCMV-HYAL2-MUT into HeLa cells and expression analysis using Luciferase assay

pCMV-Luc (control), pCMV-HYAL2-WT/pCMV-HYAL2-MUT and pRL-TK-Renilla plasmids (Promega) were used to transfect HeLa cells (ATCC, USA). Before transfection, approximately 3 × 10^5^ cells/well were plated into a six-well plate and grown using DMEM high glucose (Euroclone, Milan, Italy) supplemented with 10% heat-inactivated fetal bovine serum (Euroclone) and 1% penicillin/streptomycin (Euroclone) until ~ 70% confluence. Following 4–6 h of equilibration in the DMEM high-glucose medium without antibiotics, cells were transfected with 1 µg of each plasmid (i.e., pCMV-Luc, pCMV-HYAL2-WT/pCMV-HYAL2-MUT and pRL-TK-Renilla) using 0.5 µl of Lipofectamine LTX (Invitrogen). After 24 h of incubation, the cells were detached from the wells and assessed for Luciferase expression using Dual Luciferase Reporter Assay System and GloMax Discover Microplate Reader (Promega). Firefly luciferase results, in triplicates, was normalized against Renilla luciferase data and verified for statistical significance using Anova One-way (*p* < 0.05).

## Results

### Phenotypic features of the investigated populations

Our study included 104 adult semi-nomadic individuals living in the Reserves of Dja and Lobo in Southeast Cameroon who were enrolled during two fieldworks conducted between 2007 and 2009. Their camps were hard to reach by jeep on remote roads. The majority of the individuals was illiterate and spoke different dialects. To obtain informed consent from the subjects enrolled in the study, we relied on local interpreters—nurses. They were thus orally informed and those who gave their consent underwent clinical evaluation and blood withdrawal for both serological and genetic studies. The criteria for the enrolment of the Bantu control population were age and sex match, sympatry and clinical exclusion of phenotypically apparent diseases. On the basis of biological and cultural anthropological fieldwork experience in the investigated communities, the population was categorized as hunter-gatherers (Pygmy) or farmers (non-Pygmy). The enrolled subjects were divided in two populations constituted of 84 Baka Pygmies and 20 Bantu non-Pygmies.

We first investigated if height and weight traits exhibited significant differences between the two populations. Of all the subjects, phenotypic measurements were available for 27 Baka and 20 Bantu subjects, respectively. As summarized in Table 1, the two populations exhibited similar BMI values, while height and weight were significantly lower in the Pygmies. In particular, the mean stature of Baka Pygmies males was significantly lower, with a mean standard deviation score (SDS) of − 3.96 compared to − 1.24 in the sympatric Bantu samples (*p* < 0.05, one-way ANOVA). Interestingly, differences were less prominent in SDS for females, i.e., − 2.09 and − 1.02, for Baka Pygmies and Bantu, respectively (*p* < 0.05, one-way ANOVA).

**Table 1:** Summary statistics of principal phenotypic characteristics measured in this study

Group	Sex	*n*	Height SDS	SD	Weight SDS	SD	BMI SDS	SD	
Baka Pygmies	M	15	− 3.96	1.13	− 3.51	1.02	− 0.72	1.52	Average
			− 4.15		− 3.43		− 0.22		Median
Baka Pygmies	F	12	− 2.09	0.92	− 1.1	0.78	− 0.03	0.89	Average
			− 1.93		− 0.93		0.19		Median
Bantu	M	10	− 1.24	0.93	− 1.44	1.29	− 0.59	1.96	Average
			− 1.35		− 1.49		− 0.62		Median
Bantu	F	10	− 1.03	0.96	− 0.51	1.11	0.72	1.41	Average
			− 1.27		− 0.59		1.1		Median

Standard deviation scores (SDS) and standard deviations (SD) of the phenotypic measured traits height, weight and body mass index (BMI) in Baka Pygmies and Bantu subjects

### Whole exome sequencing (WES) analysis and functional classification of the genetic variants

**Fig. 1:**
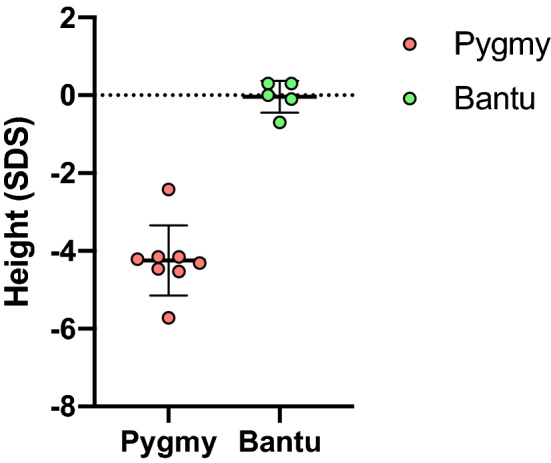
Boxplots distribution of height (SDS) measurements in Baka Pygmies and Bantu investigated subjects. Red and green circles indicated individuals subjected to WES analysis (Baka Pygmies = 8 and Bantu = 5)

**Fig. 2:**
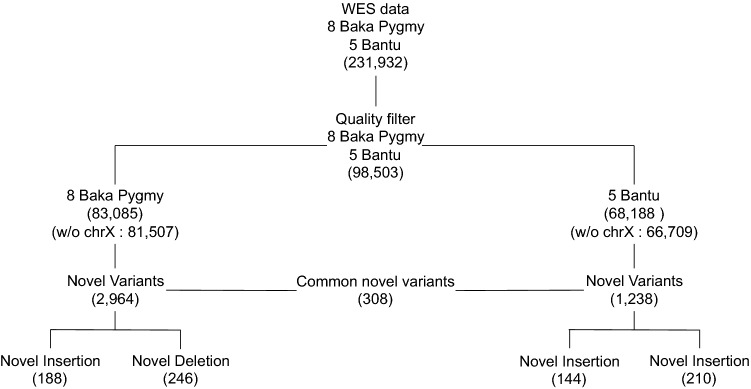
Distribution of WES variants in Baka Pygmies and Bantu investigated subjects. The numbers of variants are reported in brackets

To identify genetic variants involved in the regulation of height in the Baka Pygmies population, a WES analysis was performed. Representative samples of eight Baka Pygmies and five Bantu individuals were selected close to the lower or upper limits of their height distribution, respectively (Fig. 1). Their genomic DNA was extracted from whole blood followed by exome library preparation, next-generation sequencing (NGS) variant calling and mutation analysis. Starting from 231,932 raw variants, the application of quality control filters (see “Materials and methods”) produced 98,503 SNPs and In/Del variants (Fig. 2). Of these, 83,085 were associated with Baka Pygmies (81,507 without chromosome X) and 68,188 to Bantu samples (66,709 without chromosome X). The summary statistics of WES analysis is analytically reported in Table 2, showing the majority of variants composed by already annotated SNPs (93,365/98,503; 94.7%). Specifically, Baka Pygmies individuals displayed 78,416 SNPs (76,955 without chromosome X), and Bantu 63,905 (62,549 without chromosome X). As such, novel variants (absent in build 137 dbSNP database) were in total 7434 and 5346 for Baka Pygmies and Bantu, respectively. Of these, 3474 and 1386 were unique for the two populations.

**Table 2:** WES summary statistics

Type of variants/populations	Baka Pygmies	Bantu	Total
Number of individuals	8	5	13
Median coverage per exome	~ 16	~ 21	~ 18
Variants raw	–	–	231,932
Variants filtered (SNP/InDel)	83,085	68,188	98,503
Variants filtered SNP, InDel (w/o XY)	81,507	66,709	96,508
SNPs	78,416	63,905	93,365
SNPs (w/o XY)	76,955	62,549	91,510
Average SNPs per subject	31,413 ± 1.27	30,820 ± 0.83	–
In/Del	4669	4285	5138
In/Del (w/o XY)	4552	4158	4996
In per exome	508.75 ± 38.30	518.60 ± 28.60	–
Del per exome	566.00 ± 48.50	588.20 ± 59.70	–
Novel variants	7434	5346	8820
Novel variants unique	3474	1386	–
Novel variants unique(w/o XY)	3401	1331	–
Novel SNPs	2964	1238	3892
Novel SNPs per exome	515.25 ± 63.70	330.40 ± 39.40	–
Novel In/Del	4552	4158	5138
Novel In/Del (w/o XY)	4358	3985	4996
Novel In per exome	503.25 ± 38.00	513.60 ± 28.20	–
Novel Del per exome	562.25 ± 47.80	584.80 ± 58.30	–

Variant type and number scored in WES in Baka Pygmies and Bantu selected individuals

Remarkably, Baka Pygmies exhibited a significant increase in novel identified SNPs compared to Bantu subjects (i.e., 515.25 ± 63.7 vs. 330.4 ± 39.5, *p* = 0.014, one-way ANOVA), while numbers of novel insertions and deletions (In/Del) were roughly similar (Supplementary Table S1).

To determine the potential impact of the identified variants, the functional predictor SnpEff algorithm was employed (Table 3). As a result, 23–25% of the variants in both populations were defined as modifiers (including non-coding variants, 3′- and 5′-UTR, inter- and intra-genic variants) or variants affecting non-coding genes. Around 42% of the variants were classified as of low impact, while about 38% as of moderate impact (non-disruptive variants that might change protein effectiveness, missense variant, in-frame deletion); of note, around 1% of variants were predicted as high impact (i.e., producing truncation, or loss of protein function). In particular, Baka Pygmies exomes displayed 354 variants of high impact not shared by the control population: specifically, 30 were involved in start codon loss, 15 in stop codon loss, 148 in stop codon gain, 134 in frame-shift mutations and 27 in splice events. In contrast, Bantu exomes showed 142 high-impact variants: 12 were involved in start codon loss, three in stop codon loss, 69 in stop codon gain, 49 frame-shift mutations and 9 splice events.

**Table 3:** Functional classification of the identified genetic variants in Baka Pygmies and Bantu subjects

Populations	Baka Pygmies			Bantu		
Functional classification	Total	Novel	%	Total	Novel	%
3′UTR variant	10,577	1562	14.77	9405	1381	14.68
5′UTR prem. start codon gain	583	38	6.52	500	30	6.00
5′UTR variant	3499	531	15.18	3138	435	13.86
Disruptive inframe deletion	89	89	100.00	75	74	98.67
Disruptive inframe insertion	40	40	100.00	44	42	95.45
Downstream gene variant	1163	180	15.48	1012	156	15.42
Frameshift variant	347	343	98.85	284	282	99.30
Inframe deletion	164	164	100.00	143	142	99.30
Inframe insertion	80	80	100.00	74	71	95.95
Initiator codon variant	2	0	0.00	2	0	0.00
INTERGENIC region	33	3	9.09	28	3	10.71
Intragenic variant	1	0	0.00	1	0	0.00
Intron variant	474	73	15.40	416	55	13.22
Missense variant	29,836	2464	8.26	23,786	1514	6.37
Non coding exon variant	1064	130	12.22	853	93	10.90
Sequence feature	785	99	12.61	643	77	11.98
Splice acceptor variant	61	33	54.10	53	31	58.49
Splice donor variant	57	17	29.82	54	17	31.48
Splice region variant	1308	147	11.24	1066	120	11.26
Start lost	74	9	12.16	57	8	14.04
stop gained	286	54	18.88	235	48	20.43
Stop lost	45	2	4.44	37	2	5.41
Stop retained variant	22	2	9.09	20	1	5.00
Synonymous variant	31,011	1162	3.75	24,987	580	2.32
Upstream gene variant	1484	212	14.29	1277	179	14.02
Total	83,085	7434	8.95	68,190	5348	7.84

Functional distribution and percentage of WES variants (total and novel) found in Baka Pygmies and Bantu individuals compared with 1000 Genomes and db137 databases. Impact of variants were estimated according the SnpEff predictor

### Minor allele frequency (MAF), Fixation Index (Fst) and sparse partial least square (sPSL) regression analysis

To evaluate if MAF analysis was informative to distinguish the investigated populations, the correlation of allele frequencies for all SNPs shared between Baka Pygmies, Bantu, African and European populations was evaluated. As expected, the correlation between the Baka Pygmies and European populations was lower than between Baka Pygmies and Africans (*r*^2^ = 0.782 and *r*^2^ = 0.915, respectively). A similar trend was observed for Bantu, comparing with European (*r*^2^ = 0.774), East Asian (*r*^2^ = 0.793) and with African (*r*^2^ = 0.892) (Table 4).

**Table 4:** MAF analysis comparing Baka Pygmies and Bantu with European, African and East-Asian populations

Populations	*r*^2^	SNPs	*p*	df	CI
Baka/European	0.782	31,568	2E-16	31,566	0.778–0.786
Baka/African	0.915	31,568	2E-16	31,566	0.914–0.917
Baka/East Asian	0.764	36,255	2E-16	36,253	0.760–0.769
Bantu/European	0.774	31,568	2E-16	31,566	0.774–0.779
Bantu/African	0.892	25,213	2E-16	31,566	0.890–0.895
Bantu/East Asian	0.793	32,763	2E-16	32,761	0.789–0.797

Correlation index (*r*^2^), number of SNPs, probability (*p*), degree of freedom (df) and confidential intervals (CI) are reported

**Fig. 3:**
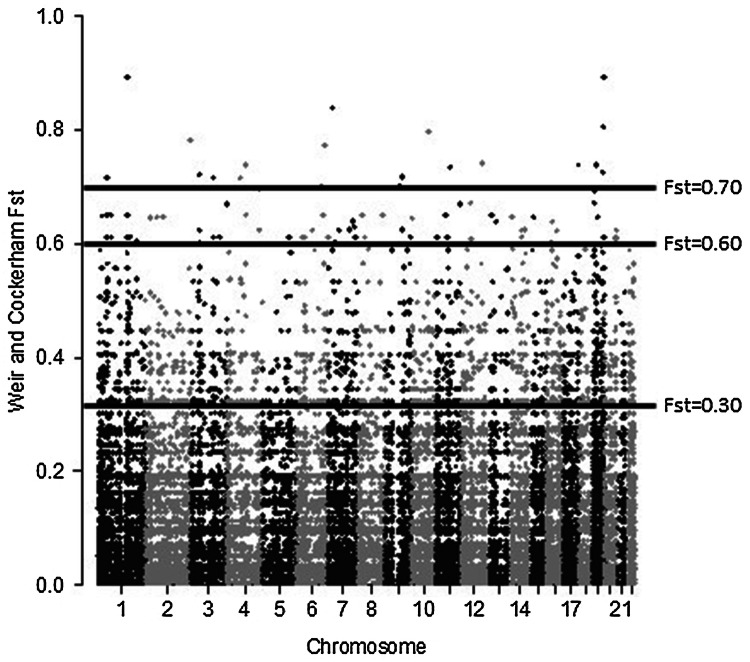
Manhattan plot Pairwise F_st_ analysis of 88,830 autosomal SNPs revealed by WES analysis. Thresholds F_st_ (i.e., 0.30, 0.60 and 0.70) were indicated

Then the likely impact and extent of genetic distance according to Wright’s population differentiation statistic, F_st_ (Pair-Wise Fixation Index) were evaluated. The determination of F_st_, starting from the entire set of 88,830 autosomal SNPs, estimated the difference in terms of genetic structure between Baka Pygmies and Bantu individuals, and assessed for each identified variant to what extent it was involved in the genetic discrimination of the two populations. Furthermore, to gain a wider insight into the extent of genetic distance, F_st_ analysis was extended to African, East-Asian and European populations included in the 1000 Genomes database, the largest public catalogue of human variation and genotype data. As a result, mean F_st_ values ranged from 0.010 to 0.380 and the weighted F_st_ from 0.030 to 0.250, indicating that the Baka Pygmies group was closer to Bantu one compared to European and East-Asian populations. In Bantu, mean F_st_ values and the weighted F_st_ showed similar trends with the other populations (Table 5). Fixing F_st_ > 0.30, 2232 SNPs significantly discriminated Baka Pygmies and Bantu samples. The most discriminating variants, corresponding to the 0.1 percentile (i.e., F_st_ > 0.60), were found in 224 genes (Fig. 3); furthermore, the 0.01 percentile cutoff (F_st_ > 0.70) highlighted 22 genes (Supplementary Table S2). Interestingly, none of these variants was previously described to be associated with Pygmies short stature studies.

**Table 5:** Genetic distances of populations according to Pair-Wise Fixation Index (F_st_)

	Baka Pygmies vs.		Bantu vs.	
	Mean F_st_	Weighted F_st_	Mean F_st_	Weighted F_st_
Bantu	0.010	0.030	–	–
AFR	0.060	0.050	0.030	0.030
EUR	0.340	0.210	0.340	0.210
EAS	0.380	0.250	0.400	0.260

F_st_ distances between different populations (*AFR* Africans, *EUR* Europeans, *EAS* East Asians)

**Fig. 4:**
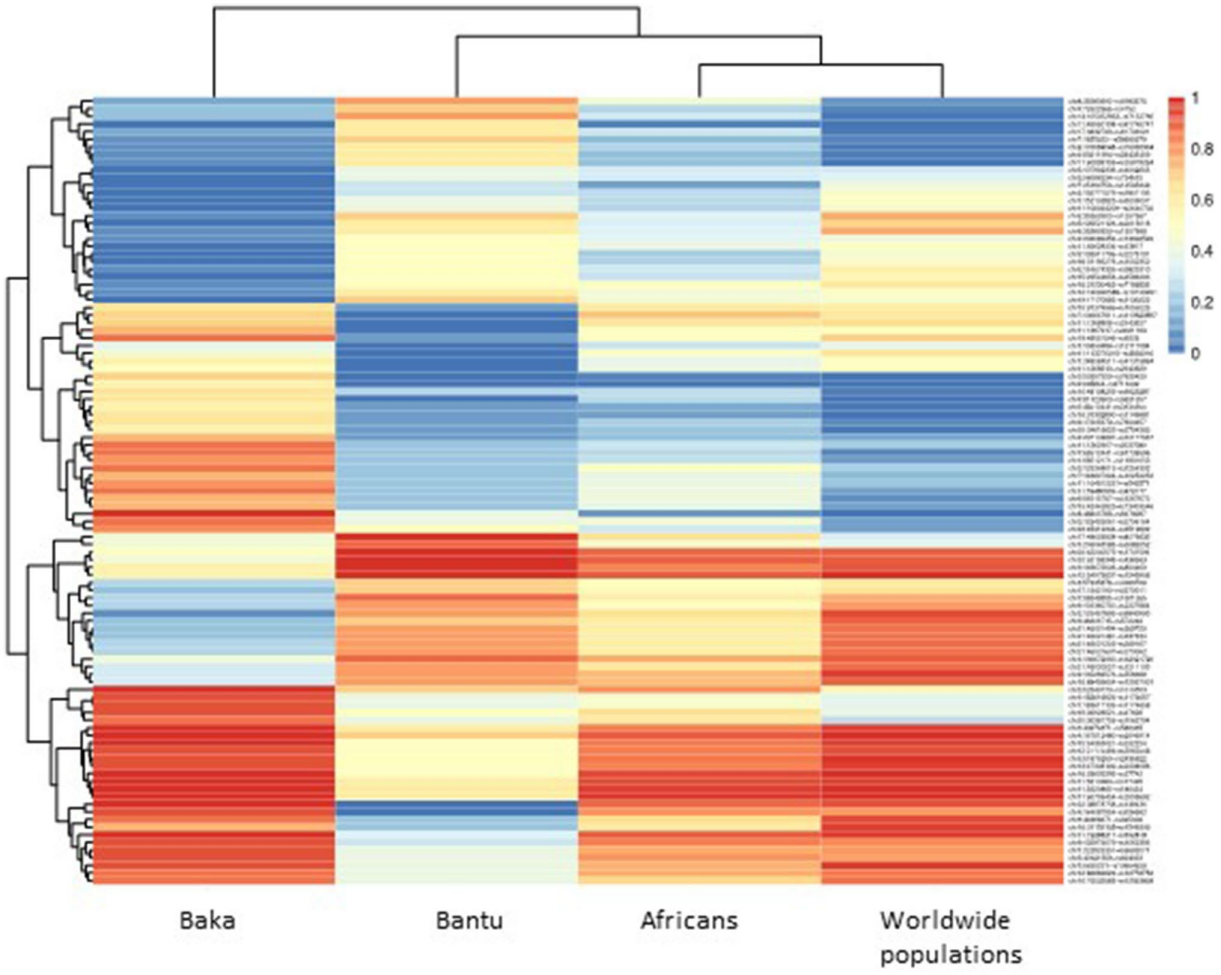
Heatmap analysis of WES variants frequency differences between Baka Pygmies, Bantu, Africans and Worldwide populations

To select the putative variants prevalently associated with Pygmies’ short stature, sPLS was applied to the genetic datasets comprising 2232 variants, previously identified using the F_st_ analysis and further reduced to 2108 bi-allelic variants. Among these, 397 variants separately clustered Baka Pygmies, compared to Bantu, African and worldwide populations (Fig. 4). Next, by hierarchical clustering, 29 SNPs associated with known genes were selected (Table 6).

**Table 6:** Gene-associated SNPs identified by hierarchical clustering

Chr	Position	SNP_ID	Gene	Ref	Alt	OR	CI_5	CI_95	*p*
16	48,138,259	rs9925287	*ABCC12*	A	G	6.257	1.988	19.7	0.001
11	59,480,969	rs472177	*OR10V1*	A	G	10.73	2.103	54.75	0.002
1	68,512,171	rs11801053	*DIRAS3*	C	T	5.995	2.054	17.5	0.001
6	46,826,715	rs572248	*GPR116*	T	A	5.02	1.644	15.32	0.004
3	48,510,441	rs3135955	*SHISA5*	G	A	5.09	1.666	15.55	0.003
1	68,512,441	rs61736596	*DIRAS3*	A	G	4.086	1.445	11.55	0.013
3	50,357,929	rs7629425	*HYAL2*	C	T	3.467	0.989	12.16	0.032
3	123,453,061	rs3796164	*MYLK*	A	G	2.929	0.609	14.09	0.653
16	31,332,890	rs1143681	*ITGAM*	C	T	2.456	0.869	6.937	0.15
11	1,262,907	rs2037089	*MUC5B*	T	C	1.754	0.538	5.718	0.745
3	123,368,013	rs1254392	*MYLK*	A	G	2.886	0.598	13.94	0.664
2	237,103,697	rs10177957	*ASB18*	G	T	1.683	0.565	5.013	0.376
15	42,442,823	rs73403546	*PLA2G4F*	G	C	1.545	0.424	5.637	0.953
4	17,805,379	rs7690457	*DCAF16*	G	A	1.567	0.55	4.466	0.565
4	81,123,603	rs6831357	*PRDM8*	G	T	1.676	0.59	4.764	0.425
2	237,103,623	rs10166966	*ASB18*	A	G	1.011	0.234	4.378	0.919
7	143,807,304	rs10252253	*OR2A2*	T	C	1.646	0.447	6.066	0.973
9	135,263,573	rs556386	*TTF1*	G	T	1.126	0.318	3.991	0.888
1	183,617,105	rs1174658	*APOBEC4*	A	G	2.207	0.58	8.401	0.65
11	104,912,221	rs542571	*CARD16*	T	A	1.245	0.412	3.765	0.915
20	34,618,622	rs2794385	*CNBD2*	G	C	1.066	0.363	3.129	1
21	46,021,481	rs437334	*KTAP10-7*	T	C	1.081	0.336	3.478	0.871
21	46,021,494	rs369720	*KTAP10-7*	A	G	1.081	0.336	3.478	0.871
16	88,495,654	rs12927001	*ZNF469*	A	G	1.848	0.405	8.422	0.933
1	183,616,926	rs1174657	*APOBEC4*	T	C	1.231	0.289	5.241	0.859
8	48,805,788	rs8178087	*PRKDC*	C	T	0.431	0.131	1.416	0.342
3	123,457,893	rs9840993	*MYLK*	G	A	0.628	0.238	1.655	0.428
20	62,198,348	rs438363	*HELZ2*	C	T	0.647	0.036	11.68	0.391
22	45,312,244	rs6519902	*PHF21B*	G	A	0.303	0.102	0.899	0.027

For 29 SNPs identified by hierarchical clustering, chromosomal coordinates, the associated genes, reference and alternative alleles, odd ratios and confidence intervals and associated probabilities are indicated

### Identification of candidate variants for pygmy short stature

**Fig. 5:**
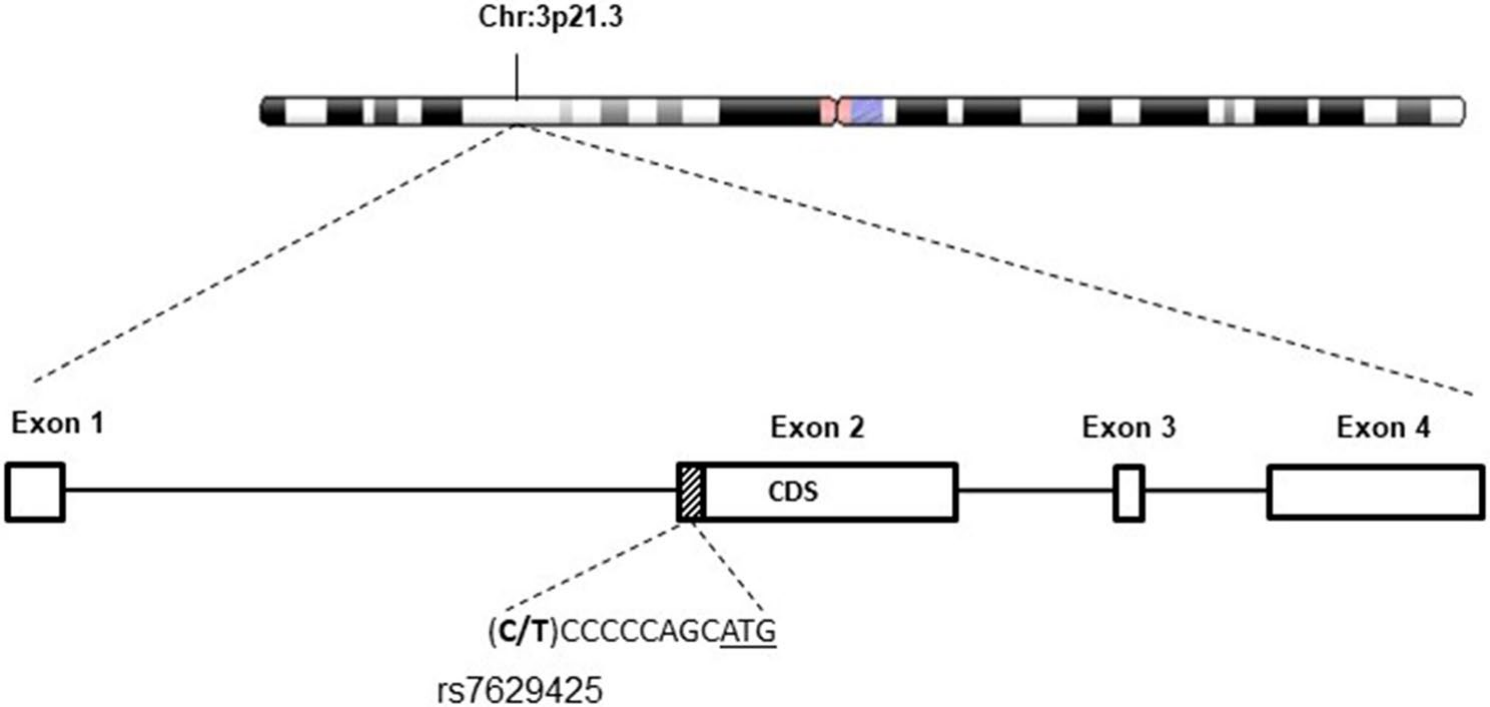
*HYAL2* chromosomal localization, gene structure and fine mapping of rs7629425 variant (in bold). ATG start codon is underlined

These 29 selected variants were then validated in a larger cohort composed of 76 Baka Pygmies and 15 Bantu individuals. To address this point, the Fluidigm 48 × 48 Access Array enrichment system was employed, highlighting that nine variants, among the 29 tested, were significantly associated with Baka Pygmies (data not shown). In particular, five variants mapped within 5′- or 3′-UTR, four in coding regions comprising two missense mutations (Table 7). Again, none of these variants has been previously associated with human height trait determination. A promising variant, significantly associated with short stature in Baka individuals (*p* = 0.032, Chi-Square, Table 6) was rs7629425, a C/T SNP located in the 5′-UTR of *HYAL2* gene, nine nucleotides upstream of exon two coding sequence (Fig. 5).

**Table 7:** Main candidate variants associated with Baka Pygmies’ short stature

Gene	SNP ID	Localization	MAF (Baka Pygmies)	MAF (African)	Frequency difference
*SHISA5*	rs3135955	3′-UTR	0.570	0.130	0.440
*HYAL2*	rs7629425	5′-UTR	0.440	0.030	0.410
*ABCC12*	rs9925287	CDS	0.620	0.300	0.320
*DIRAS3*	rs11801053	3′-UTR	0.590	0.270	0.320
*DIRAS*	rs61736596	CDS	0.550	0.280	0.270
*STX3*	rs472177	5′-UTR	0.650	0.480	0.170
*PLA2G4C*	rs9226	3′-UTR	0.650	0.500	0.150
*DBT*	rs12021720	CDS	0.320	0.250	0.070
*GPR116*	rs572248	CDS	0.620	0.550	0.070

For each SNPs validated by Fluidigm 48 × 48 access array enrichment system, the genes localizations and MAF frequencies between Baka and African populations are indicated

The C to T nucleotide variation was predicted to affect Sp3 or Rfx1 transcription binding, respectively (PROMO 3.0 https://alggen.lsi.upc.es/, data not shown). In particular, Sp3 transcription factor was reported to affect several biological processes including ossification (Göllner et al. 2001). Then, a luciferase reporter assay was performed, comparing transcriptional activities of the two alleles through eukaryotic expression vectors (see “Materials and methods”). After HeLa cells transfections with allele-specific plasmids, normalized luciferase measurements at 24 h post-treatment (p.t.) indicated a significant increase in the gene reporter expression in correspondence of the alternative T allele compared to the reference one (*n* = 6, *p* = 0.024, one-way ANOVA).

## Discussion

In the present contribution, we describe the results of a WES analysis of individuals from two Cameroonians populations, specifically hunter-gatherers Baka Pygmies and their neighbor Bantu non-Pygmies farmers in an attempt to identify genetic variants associated with short stature.

Previous studies have collected clinical and biological data of Pygmies, related to their lifestyle, culture and environment and various groups identified candidate genes contributing to their short stature phenotype (Lachance et al. 2012). These are in particular *HESX1* (which encodes a homeobox containing transcriptional repressor that plays a critical role in development of the anterior pituitary, the site of growth hormone synthesis and secretion), *APPL1* (which is involved in crosstalk between adiponectin and insulin signaling pathways), *ASB14* (which encodes a SOCS box protein), and the sperm-motility gene *DNAH12*. However, the causative mechanisms for Pygmies’ short stature still remain a debated topic. Moreover, from a clinical point of view, understanding the molecular mechanisms involved in Pygmies’ short stature is important not only from an evolutionary point of view, but also because it could provide additional information regarding a potential novel cause of children with idiopathic short stature (Kang 2017).

In a WES genetic approach identifying genes associated with short stature in Baku Pygmies, a primary aspect to consider is the inclusion of a proper control population for data comparison. To this purpose, while Baku is a relatively homogeneous population relying on an economy based on hunting and gathering, it also has a complex socioeconomic relationship with the farming neighbor population, known as Bantu (Rozzi et al. 2015). From an evolutionary point of view, Baka Pygmies and Bantu populations shared a common ancestor, but started diverging as separate populations around 60,000 years ago. However, substantial admixtures between these populations have occurred in the last 5–1000 years (Perry et al. 2014).

Starting from the genetic material obtained from phenotypically selected Baka Pygmies and Bantu individuals, we performed Pair-Wise Fixation Index and sparse partial least square regression method analysis for positive selection of variants associated with short stature. Following gene prioritization, validation of initial reads in a larger cohort of individuals and functional annotation of candidate genes harboring genetic variants enriched in the Baka Pygmies population, we hypothesized that the identified rs7629425 variation, located in the 5′-UTR region of Hyaluronoglucosaminidase 2 (*HYAL2*) gene, might have a role in the determination of short height in Pygmies. *HYAL2* encodes for a GPI-anchored cell surface protein that degrades hyaluronan, one of the major glycosaminoglycans of the extracellular matrix. Hyaluronan and its degradation fragments are thought to be involved in cell proliferation, migration and differentiation (Lepperdinger et al. 2001). Furthermore, the gene encodes two alternatively spliced transcript variants, which differ only in the 5′-UTR, supporting the idea that this region may have an important regulatory function on gene transcription. *HYAL2* depletion in conditional knockout mice showed that this gene was essential for the catabolism of hyaluronan. In the absence of *HYAL2*, extracellular hyaluronan accumulated and, in some cases, may lead to cardiopulmonary dysfunctions (Chowdhury et al. 2013). In a different study, it has been reported that mice lacking *HYAL2* displayed variably penetrant developmental defects, including skeletal and cardiac anomalies (Triggs-Raine and Natowicz 2015). Importantly, *HYAL2* is localized on human chromosome region 3p21.3 described to be associated with short stature in Pygmies, containing loci associated with growth hormone, insulin and insulin-like growth factor signaling pathways, as well as immunity and neuroendocrine signaling involved in reproduction and metabolism (Jarvis et al. 2012). This region also includes the positional candidate gene *DOCK3*, which is known to be associated with height variation in Europeans, and *CISH*, a negative regulator of cytokine signaling known to inhibit growth hormone-stimulated STAT5 signaling (Jarvis et al. 2012). The 5′-UTR localization of rs7629425, nine nucleotides upstream of the first Methionine residue, was hypothesized to differently affect the binding of transcription factors. To functionally evaluate the effects of C–T variation in regulating transcription, a reporter Luciferase assay was performed indicating a significant and most prominent transcriptional activity for the alternative T allele.

In summary, our results indicate the importance of a WES approach to generate data for identifying functionally important genetic variants associated with complex traits like stature in humans. In particular, we found enrichment of a rare variant in the non-coding 5′-UTR region of *HYAL2* gene in the Baka Pygmies population, in a chromosomal region previously described to be linked to this peculiar phenotype. Future studies will be devoted to further investigate the molecular effects of the identified nucleotide variant on the cascade of genes involved in the regulation of body stature.

## Electronic supplementary material

Below is the link to the electronic supplementary material.

Supplementary file1 (JPG 32 kb)

Supplementary file2 (DOCX 18 kb)

## References

[CR1] Andrade AC, Jee YH, Nilsson O (2017). New genetic diagnoses of short stature provide insights into local regulation of childhood growth. Horm Res Paediatr.

[CR2] Bailey RC (1991). The comparative growth of Efe Pygmies and African farmers from birth to age 5 years. Ann Hum Biol.

[CR3] Becker NS, Verdu P, Froment A, Le Bomin S, Pagezy H, Bahuchet S, Heyer E (2011). Indirect evidence for the genetic determination of short stature in African Pygmies. Am J Phys Anthropol.

[CR4] Bozzola M, Travaglino P, Marziliano N, Meazza C, Pagani S, Grasso M, Tauber M, Diegoli M, Pilotto A, Disabella E, Tarantino P, Brega A, Arbustini E (2009). The shortness of Pygmies is associated with severe under-expression of the growth hormone receptor. Mol Genet Metab.

[CR5] Chowdhury B, Hemming R, Hombach-Klonisch S, Flamion B, Triggs-Raine B (2013). Murine hyaluronidase 2 deficiency results in extracellular hyaluronan accumulation and severe cardiopulmonary dysfunction. J Biol Chem.

[CR6] Chun H, Keleş S (2010). Sparse partial least squares regression for simultaneous dimension reduction and variable selection. J R Stat Soc Series B Stat Methodol.

[CR7] Cohen LE (2014). Idiopathic short stature: a clinical review. JAMA.

[CR8] Durand C, Rappold GA (2013). Height matters-from monogenic disorders to normal variation. Nat Rev Endocrinol.

[CR9] Garrison E, Marth G (2012). Haplotype-based variant detection from short-read sequencing. arXiv.

[CR10] Göllner H, Dani C, Phillips B, Philipsen S, Suske G (2001). Impaired ossification in mice lacking the transcription factor Sp3. Mech Dev.

[CR11] Hsieh P, Veeramah KR, Lachance J, Tishkoff SA, Wall JD, Hammer MF, Gutenkunst RN (2016). Whole-genome sequence analyses of Western Central African Pygmy hunter-gatherers reveal a complex demographic history and identify candidate genes under positive natural selection. Genome Res.

[CR12] Jarvis JP, Scheinfeldt LB, Soi S, Lambert C, Omberg L, Ferwerda B, Froment A, Bodo JM, Beggs W, Hoffman G, Mezey J, Tishkoff SA (2012). Patterns of ancestry, signatures of natural selection, and genetic association with stature in Western African Pygmies. PLoS Genet.

[CR13] Kang MJ (2017). Novel genetic cause of idiopathic short stature. Ann Pediatr Endocrinol Metab.

[CR14] Lachance J, Vernot B, Elbers CC, Ferwerda B, Froment A, Bodo JM, Lema G, Fu W, Nyambo TB, Rebbeck TR, Zhang K, Akey JM, Tishkoff SA (2012). Evolutionary history and adaptation from high-coverage whole-genome sequences of diverse African hunter-gatherers. Cell.

[CR15] Le Bouc Y (2017). Have we finally solve the enigma of the small size of Pygmies?. Ann Endocrinol.

[CR16] Lepperdinger G, Müllegger J, Kreil G (2001). Hyal2-less active, but more versatile?. Matrix Biol.

[CR17] Lettre G (2011). Recent progress in the study of the genetics of height. Hum Genet.

[CR18] Li H, Durbin R (2010). Fast and accurate long-read alignment with Burrows-Wheeler transform. Bioinformatics.

[CR19] Meazza C, Pagani S, Bozzola M (2011). The pygmy short stature enigma. Pediatr Endocrinol Rev.

[CR20] Mendizabal I, Marigorta UM, Lao O, Comas D (2012). Adaptive evolution of loci covarying with the human African Pygmy phenotype. Hum Genet.

[CR21] Migliano AB, Vinicius L, Lahr MM (2007). Life history trade-offs explain the evolution of human Pygmies. Proc Natl Acad Sci USA.

[CR22] Patin E, Siddle KJ, Laval G, Quach H, Harmant C, Becker N, Froment A, Régnault B, Lemée L, Gravel S, Hombert JM, Van der Veen L, Dominy NJ, Perry GH, Barreiro LB, Verdu P, Heyer E, Quintana-Murci L (2014). The impact of agricultural emergence on the genetic history of African rainforest hunter-gatherers and agriculturalists. Nat Commun.

[CR23] Pemberton TJ, Verdu P, Becker NS, Willer CJ, Hewlett BS, Le Bomin S, Froment A, Rosenberg NA, Heyer E (2018). A genome scan for genes underlying adult body size differences between Central African hunter-gatherers and farmers. Hum Genet.

[CR24] Perry GH, Dominy NJ (2009). Evolution of the human pygmy phenotype. Trends Ecol Evol.

[CR25] Perry GH, Foll M, Grenier JC, Patin E, Nédélec Y, Pacis A, Barakatt M, Gravel S, Zhou X, Nsobya SL, Excoffier L, Quintana-Murci L, Dominy NJ, Barreiro LB (2014). Adaptive, convergent origins of the pygmy phenotype in African rainforest hunter-gatherers. Proc Natl Acad Sci USA.

[CR26] Rozzi FV, Koudou Y, Froment A, Le Bouc Y, Botton J (2015). Growth pattern from birth to adulthood in African Pygmies of known age. Nat Commun.

[CR27] Sohail M, Maier RM, Ganna A, Bloemendal A, Martin AR, Turchin MC, Chiang CW, Hirschhorn J, Daly MJ, Patterson N, Neale B, Mathieson I, Reich D, Sunyaev SR (2019). Polygenic adaptation on height is overestimated due to uncorrected stratification in genome-wide association studies. Elife.

[CR28] Triggs-Raine B, Natowicz MR (2015). Biology of hyaluronan: insights from genetic disorders of hyaluronan metabolism. World J Biol Chem.

[CR29] Verdu P (2016). African Pygmies. Curr Biol.

[CR30] Verdu P, Austerlitz F, Estoup A, Vitalis R, Georges M, Théry S, Froment A, Le Bomin S, Gessain A, Hombert JM, Van der Veen L, Quintana-Murci L, Bahuchet S, Heyer E (2009). Origins and genetic diversity of Pygmy hunter-gatherers from western Central Africa. Curr Biol.

[CR31] Waldman LA, Chia DJ (2013). Towards identification of molecular mechanisms of short stature. Int J Pediatr Endocrinol.

[CR32] Wit JM, Clayton PE, Rogol AD, Savage MO, Saenger PH, Cohen P (2008). Idiopathic short stature: definition, epidemiology, and diagnostic evaluation. Growth Horm IGF Res.

[CR33] Wit JM, Oostdijk W, Losekoot M, van Duyvenvoorde HA, Ruivenkamp CA, Kant SG (2016). Mechanisms in endocrinology: novel genetic causes of short stature. Eur J Endocrinol.

[CR34] Wood AR, Esko T, Yang J, Vedantam S, Pers TH, Gustafsson S, Chu AY, Estrada K, Luan J, Kutalik Z, Amin N, Buchkovich ML, Croteau-Chonka DC, Day FR, Duan Y, Fall T, Fehrmann R, Ferreira T, Jackson AU, Karjalainen J, Lo KS, Locke AE, Mägi R, Mihailov E, Porcu E, Randall JC, Scherag A, Vinkhuyzen AA, Westra HJ, Winkler TW, Workalemahu T, Zhao JH, Absher D, Albrecht E, Anderson D, Baron J, Beekman M, Demirkan A, Ehret GB, Feenstra B, Feitosa MF, Fischer K, Fraser RM, Goel A, Gong J, Justice AE, Kanoni S, Kleber ME, Kristiansson K, Lim U, Lotay V, Lui JC, Mangino M, Mateo Leach I, Medina-Gomez C, NallsMA NDR, Palmer CD, PaskoD PS, Prokopenko I, Ried JS, Ripke S, Shungin D, Stancáková A, Strawbridge RJ, Sung YJ, Tanaka T, Teumer A, Trompet S, van der Laan SW, van Setten J, Van Vliet-Ostaptchouk JV, Wang Z, YengoL ZW, Afzal U, Arnlöv J, Arscott GM, Bandinelli S, Barrett A, Bellis C, Bennett AJ, Berne C, Blüher M, Bolton JL, Böttcher Y, Boyd HA, Bruinenberg M, Buckley BM, Buyske S, CaspersenIH CPS, Clarke R, Claudi-Boehm S, Cooper M, Daw EW, De Jong PA, Deelen J, Delgado G, Denny JC, Dhonukshe-Rutten R, Dimitriou M, Doney AS, Dörr M, Eklund N, Eury E, Folkersen L, Garcia ME, Geller F, Giedraitis V, Go AS, Grallert H, Grammer TB, Gräßler J, Grönberg H, de Groot LC, Groves CJ, Haessler J, Hall P, Haller T, Hallmans G, Hannemann A, Hartman CA, Hassinen M, Hayward C, Heard-Costa NL, Helmer Q, Hemani G, Henders AK, Hillege HL, Hlatky MA, Hoffmann W, Hoffmann P, Holmen O, Houwing-Duistermaat JJ, Illig T, Isaacs A, James AL, Jeff J, Johansen B, Johansson Å, Jolley J, JuliusdottirT JJ, Kho AN, KinnunenL KN, Kocher T, Kratzer W, Lichtner P, Lind L, Lindström J, Lobbens S, Lorentzon M, Lu Y, Lyssenko V, Magnusson PK, Mahajan A, Maillard M, McArdle WL, McKenzie CA, McLachlan S, McLaren PJ, Menni C, Merger S, Milani L, Moayyeri A, Monda KL, Morken MA, Müller G, Müller-Nurasyid M, Musk AW, Narisu N, Nauck M, Nolte IM, Nöthen MM, Oozageer L, Pilz S, Rayner NW, Renstrom F, Robertson NR, Rose LM, Roussel R, Sanna S, Scharnagl H, Scholtens S, Schumacher FR, SchunkertH SRA, Sehmi J, Seufferlein T, Shi J, SilventoinenK SJH, Smith AV, Smolonska J, Stanton AV, Stirrups K, Stott DJ, Stringham HM, Sundström J, SwertzMA SAC, Tayo BO, Thorleifsson G, Tyrer JP, van Dijk S, van Schoor NM, van der Velde N, van Heemst D, van Oort FV, Vermeulen SH, Verweij N, VonkJM WLL, Waldenberger M, Wennauer R, Wilkens LR, Willenborg C, Wilsgaard T, WojczynskiMK WA, Wright AF, Zhang Q, Arveiler D, Bakker SJ, Beilby J, Bergman RN, Bergmann S, Biffar R, Blangero J, Boomsma DI, Bornstein SR, Bovet P, Brambilla P, Brown MJ, Campbell H, Caulfield MJ, Chakravarti A, Collins R, Collins FS, Crawford DC, Cupples LA, Danesh J, de Faire U, den Ruijter HM, Erbel R, Erdmann J, Eriksson JG, Farrall M, Ferrannini E, Ferrières J, Ford I, Forouhi NG, Forrester T, Gansevoort RT, Gejman PV, Gieger C, Golay A, Gottesman O, GudnasonV GU, Haas DW, Hall AS, Harris TB, Hattersley AT, Heath AC, Hengstenberg C, Hicks AA, Hindorff LA, Hingorani AD, Hofman A, Hovingh GK, Humphries SE, Hunt SC, Hypponen E, Jacobs KB, Jarvelin MR, Jousilahti P, Jula AM, Kaprio J, Kastelein JJ, Kayser M, Kee F, Keinanen-Kiukaanniemi SM, Kiemeney LA, Kooner JS, Kooperberg C, Koskinen S, Kovacs P, Kraja AT, Kumari M, Kuusisto J, Lakka TA, Langenberg C, Le Marchand L, Lehtimäki T, Lupoli S, Madden PA, MännistöS MP, Marette A, Matise TC, McKnight B, Meitinger T, Moll FL, Montgomery GW, Morris AD, Morris AP, Murray JC, Nelis M, Ohlsson C, Oldehinkel AJ, Ong KK, Ouwehand WH, Pasterkamp G, Peters A, Pramstaller PP, Price JF, Qi L, RaitakariOT RT, Rao DC, Rice TK, Ritchie M, Rudan I, Salomaa V, Samani NJ, Saramies J, Sarzynski MA, Schwarz PE, Sebert S, Sever P, Shuldiner AR, SinisaloJ SV, Stolk RP, Tardif JC, Tönjes A, Tremblay A, Tremoli E, Virtamo J, Vohl MC, Amouyel P, Asselbergs FW, Assimes TL, Bochud M, Boehm BO, Boerwinkle E, Bottinger EP, Bouchard C, Cauchi S, Chambers JC, Chanock SJ, Cooper RS, de Bakker PI, Dedoussis G, Ferrucci L, Franks PW, Froguel P, Groop LC, Haiman CA, Hamsten A, Hayes MG, Hui J, Hunter DJ, Hveem K, Jukema JW, Kaplan RC, Kivimaki M, Kuh D, Laakso M, Liu Y, Martin NG, März W, Melbye M, Moebus S, Munroe PB, Njølstad I, Oostra BA, Palmer CN, Pedersen NL, Perola M, Pérusse L, Peters U, Powell JE, Power C, Quertermous T, Rauramaa R, Reinmaa E, Ridker PM, Rivadeneira F, Rotter JI, Saaristo TE, Saleheen D, Schlessinger D, SlagboomPE SH, Spector TD, Strauch K, Stumvoll M, Tuomilehto J, Uusitupa M, van der Harst P, Völzke H, Walker M, Wareham NJ, Watkins H, Wichmann HE, Wilson JF, Zanen P, Deloukas P, Heid IM, Lindgren CM, Mohlke KL, Speliotes EK, Thorsteinsdottir U, Barroso I, Fox S, North KE, Strachan DP, Beckmann JS, Berndt SI, Boehnke M, Borecki IB, McCarthy MI, Metspalu A, Stefansson K, Uitterlinden AG, van Duijn CM, Franke L, Willer CJ, Price AL, Lettre G, Loos RJ, Weedon MN, Ingelsson E, O'Connell JR, Abecasis GR, Chasman DI, Goddard ME, Visscher PM, Hirschhorn JN, Frayling TM, Electronic Medical Records and Genomics (eMEMERGEGE) Consortium; MIGen Consortium; PAGEGE Consortium; LifeLines Cohort Study (2014). Defining the role of common variation in the genomic and biological architecture of adult human height. Nat Genet.

[CR35] Wright S (1951). The genetical structure of populations. Ann Eugen.

