# Supplementary figures and images for: Identification of novel genetic variants associated with short stature in a Baka Pygmies population
Source: Hum Genet. 2020 Jun 24;139(11):1471–83. doi: 10.1007/s00439-020-02191-x (PMC7519921; doi:10.1007/s00439-020-02191-x)

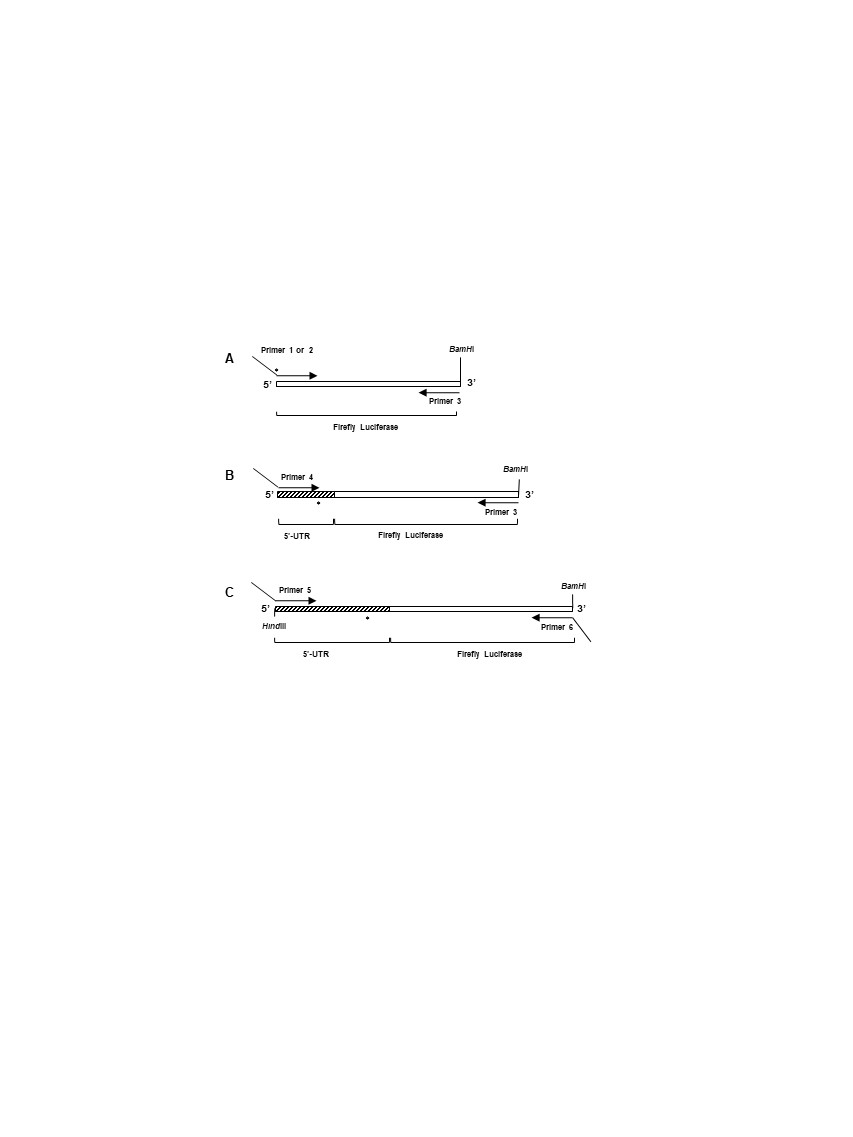

Supplement: Supplementary file 1 — Supplementary file1 (JPG 32 kb) [file 439_2020_2191_MOESM1_ESM.jpg]
