# Supplementary material for: Identification of novel genetic variants associated with short stature in a Baka Pygmies population
Source: Hum Genet. 2020 Jun 24;139(11):1471–83. doi: 10.1007/s00439-020-02191-x (PMC7519921; doi:10.1007/s00439-020-02191-x)
Supplement: Supplementary file 2 — Supplementary file2 (DOCX 18 kb) [file 439_2020_2191_MOESM2_ESM.docx]

**Supplementary Table S1** Distribution of novel genetics variants (SNPs, insertions and deletions) in the investigated Baka Pygmies and Bantu subjects

| **Baka Pygmies** |  |  |  |  |  |  |  |  |  |  |
| --- | --- | --- | --- | --- | --- | --- | --- | --- | --- | --- |
| Samples | 1 | 2 | 3 | 4 | 5 | 6 | 7 | 8 | Total | Average |
| SNPs | 582 | 541 | 514 | 377 | 535 | 525 | 566 | 482 | 4122 | 515.25±63.7 |
| Ins | 535 | 509 | 503 | 466 | 528 | 556 | 490 | 439 | 4026 | 503.25±38 |
| Dels | 591 | 577 | 601 | 487 | 571 | 621 | 552 | 498 | 4498 | 562.25±47.7 |
|  |  |  |  |  |  |  |  |  |  |  |
| **Bantu** |  |  |  |  |  |  |  |  |  |  |
| Samples | 1 | 2 | 3 | 4 | 5 | Total |  |  |  | Average |
| SNPs | 298 | 333 | 361 | 284 | 376 | 1652 |  |  |  | 330.4±39.5 |
| Ins | 515 | 541 | 532 | 512 | 468 | 2568 |  |  |  | 513±28 |
| Dels | 600 | 660 | 583 | 584 | 497 | 2924 |  |  |  | 584.8±58.3 |

**Supplementary Table S2** F_st_ analysis ranking of WES identified variants

| **Chr** | ***P*** | **ID** | **Gene** | **F_st_** |
| --- | --- | --- | --- | --- |
| 1 | 1.6E+08 | rs147713038 | *MSTO1* | 0.89 |
| 19 | 5.8E+07 | rs111313171 | *ZNF814* | 0.89 |
| 7 | 2.2E+07 | rs10950854 | *DNAH11* | 0.84 |
| 19 | 5.4E+07 | rs74429916 | *VN1R4* | 0.81 |
| 10 | 9.1E+07 | rs12242568 | *IFIT3* | 0.80 |
| 2 | 2.4E+08 | - | *GPC1* | 0.78 |
| 6 | 1.5E+08 | rs146950621 | *ULBP2* | 0.77 |
| 12 | 1.1E+08 | rs2287557 | *TMEM119* | 0.74 |
| 17 | 7.9E+07 | rs35572189 | *BAHCC1* | 0.74 |
| 19 | 1.7E+07 | rs1130222 | *HAUS8* | 0.74 |
| 4 | 1E+08 | rs17029090 | *C4orf17* | 0.74 |
| 11 | 7.2E+07 | rs392818 | *PDE2A* | 0.73 |
| 19 | 4.9E+07 | rs9226 | *PLA2G4C* | 0.73 |
| 3 | 4.8E+07 | rs9847953 | *ZNF589* | 0.72 |
| 9 | 9.1E+07 | rs7048023 | *RP13-60M5.2* | 0.72 |
| 9 | 9.1E+07 | rs28550932 | *RP13-60M5.2* | 0.72 |
| 1 | 4.5E+07 | rs3820586 | *KIF2C* | 0.72 |
| 3 | 1.2E+08 | rs9840993 | *MYLK* | 0.72 |
| 4 | 7E+07 | - | *UGT2B7* | 0.72 |
| 6 | 1.4E+08 | rs2297339 | *HBS1L* | 0.70 |
| 9 | 7.9E+07 | rs3739729 | *RFK* | 0.70 |
| 4 | 1.7E+08 | rs59621536 | *HAND2* | 0.70 |

Variants priorization was obtained fixing Fst>0.70. Chromosome

(Chr), probability (P), SNPs identification (ID) and F_st_ vales are

reported.

**Supplementary Table S3.** Primers used for generate and sequencing of pCMV-HYAL2-WT

and pCMV-HYAL2-MUT plasmids.

| **Primer** | **Sequence (5’-3’)** |
| --- | --- |
| 1 | AGGTGACACCTCCTGCAGCCCCCAGCATGGAAGACGCCAAAAA |
| 2 | AGGTGACACCTCCTGCAACCCCCAGCATGGAAGACGCCAAAAA |
| 3 | AAGTGGATCCTTACACGGCGATCTTTCCGCCCTTCTTGGC |
| 4 | GCAAAGCTTGCGAGTTCCTGAGCTGGTGCCAGGCAGGTGACACCTCCT |
| 5 | GCGCATGCAAAGCTTGCGAGTTCCTGAGCT |
| 6 | CTCGCCAAGTGGATCCTTACACGGCGATCT |
| Luc 1 | CGGGAGGTAGATGAGATGTGA |
| Luc 3 | GTTATGTAAACAATCCGGAAGCG |
